# Identification of Differentially Expressed Genes in Pituitary Adenomas by Integrating Analysis of Microarray Data

**DOI:** 10.1155/2015/164087

**Published:** 2015-01-06

**Authors:** Peng Zhao, Wei Hu, Hongyun Wang, Shengyuan Yu, Chuzhong Li, Jiwei Bai, Songbai Gui, Yazhuo Zhang

**Affiliations:** ^1^Department of Neurosurgery, Beijing Tiantan Hospital, Capital Medical University, Beijing, China; ^2^Department of Cardiology, Beijing Chuiyangliu Hospital, Beijing, China; ^3^Beijing Neurosurgical Institute, Center of Brain Tumor, Beijing Institute for Brain Disorders, Capital Medical University, Beijing, China

## Abstract

Pituitary adenomas, monoclonal in origin, are the most common intracranial neoplasms. Altered gene expression as well as somatic mutations is detected frequently in pituitary adenomas. The purpose of this study was to detect differentially expressed genes (DEGs) and biological processes during tumor formation of pituitary adenomas. We performed an integrated analysis of publicly available GEO datasets of pituitary adenomas to identify DEGs between pituitary adenomas and normal control (NC) tissues. Gene function analysis including Gene Ontology (GO), Kyoto Encyclopedia of Genes and Genomes (KEGG) pathway enrichment analysis, and protein-protein interaction (PPI) networks analysis was conducted to interpret the biological role of those DEGs. In this study we detected 3994 DEGs (2043 upregulated and 1951 downregulated) in pituitary adenoma through an integrated analysis of 5 different microarray datasets. Gene function analysis revealed that the functions of those DEGs were highly correlated with the development of pituitary adenoma. This integrated analysis of microarray data identified some genes and pathways associated with pituitary adenoma, which may help to understand the pathology underlying pituitary adenoma and contribute to the successful identification of therapeutic targets for pituitary adenoma.

## 1. Introduction

Pituitary adenomas account for 10–15% of all intracranial neoplasms. Most pituitary adenomas are benign, although they may cause significant morbidity through mass effects and/or the improper secretion of pituitary hormones, indicating that the development of pituitary adenomas is a complex multistep process. Pituitary adenomas usually occur sporadically and are grouped into functioning and nonfunctioning adenomas (NFAs) according to hormonal status and further subdivided into microadenomas (<1 cm) and macroadenomas (≥1 cm) based on tumor size [1].

Despite massive research, the pathogenesis of pituitary adenomas still remains unclear. However, advances in molecular biology such as microarray technique enable the identification of new genes associated with pituitary tumor genesis. The microarray technique, which allows the simultaneous analysis of thousands of genes at the transcript expression level in a single experiment [2], has greatly facilitated the investigation of gene expression differences between normal pituitary and pituitary adenomas. Recently, researchers have used this powerful technique to compare gene expression between normal pituitary tissues and pituitary adenomas of different origins and have also identified many genes associated with certain tumor types [3–6]. However, there are inconsistencies among these studies due to limitations of different sample sources, microarray platforms, and analysis techniques [7]. Towards this end, we performed a systematic integration of gene expression data from multiple sources, to increase statistical power for detecting differentially expressed genes (DEGs) [8, 9]. Now in this study we use this method to identify DEGs and biological processes associated with pituitary adenomas to provide some insights into molecular mechanisms underlying the pathogenesis of pituitary adenomas and many guided further therapies for this disease.

## 2. Material and Methods

### 2.1. Identification of Eligible Gene Expression Datasets

Expression profiling studies of pituitary adenomas were identified by searching the Gene Expression Omnibus database (GEO, http://www.ncbi.nlm.nih.gov/geo/) [12]. We only collected original experimental articles that analyzed gene expression profiling between pituitary adenoma and normal control (NC) tissues. Nonhuman studies, review articles, and integrated analysis of expression profiles were excluded.

### 2.2. Data Preprocessing

Normalization is very important to compare multiple microarray datasets accurately. The heterogeneity of multiple datasets resulted from different platforms, and clinical samples may make it difficult to compare the results directly. Consequently a global normalization approach should be included to minimize the heterogeneity. For this purpose, we first preprocessed the raw microarray data of each study by log2 transformation, then the Z-score transformation was applied for calculation of expression intensities of each probe, and Z-scores were calculated following the formula

(1) $$\begin{array}{c} \text{Z}\;\text{score}=\frac{x_{i}-\overset{-}{x}}{\delta }, \end{array}$$

where *x*_*i*_ indicates raw intensity data for each gene; $\overset{-}{x}$ indicates the average intensity of the gene in a single experiment, and *δ* indicates standard deviation (SD) of all the measured intensities.

### 2.3. Statistical Analysis

The significance analysis of microarray (SAM) software was used to determine the DEGs between pituitary adenoma and NC tissues. Gene specific *t*-tests were carried out, outputting a “relative difference” score or *d* value which was defined as the average expression change of each gene from different expression levels to the SD of measurements. The genes with at least 1.5-fold change and a false discovery rate (FDR) less than 0.05 were selected as DEGs [13].

### 2.4. Functional Annotation of DEGs

To interpret the biological functions of the DEGs, we performed Gene Ontology (GO) enrichment analysis to explore functional distribution of DEGs in pituitary adenoma. GO provides a common descriptive framework and functional annotation of the gene sets data. Furthermore we also performed Kyoto Encyclopedia of Genes and Genomes (KEGG) pathway enrichment analysis for DEGs to find important pathways involved in pituitary adenoma. KEGG pathway database is a recognized and comprehensive database including all kinds of biochemistry pathways [14]. The online based software GENECODIS was utilized in this analysis [15].

### 2.5. PPI Network Construction

The protein-protein interactions (PPIs) analysis was conducted to investigate the functions of proteins at the molecular level [16]. The identification of protein interactions in a genome-wide scale is important to uncover the cellular regulation mechanisms [17]. Biological General Repository for Interaction Datasets (BioGRID) (http://thebiogrid.org/) was used to construct the PPI network, and then Cytoscape software was used to visualize the distribution characteristics of the top 10 up- and downregulated DEGs in the PPI network [18].

## 3. Results

### 3.1. Short Overview of the Studies Included

In this study, we obtained a total of 5 expression profiles of pituitary adenoma in GEO database; it contained 44 samples of pituitary adenoma and 12 samples of controls. The individual studies for analyzing are displayed in Table 1. Several types of pituitary adenomas were included in our study such as NFPA, growth hormone pituitary adenomas, and prolactin adenomas.

### 3.2. Detecting Genes Associated with Pituitary Adenoma

After global normalization, we adopted SAM software to identify DEGs between pituitary adenomas and control samples. With FDR ≤0.05 and a minimal fold change of 1.5, a total of 3994 genes were found to show aberrant expression in samples of pituitary adenoma compared with NC tissues, among which 2043 DEGs were upregulated and 1951 were downregulated. A list of the top 10 most significantly up- or downregulated genes was presented in Table 2.

The upregulated gene with the lowest *P* value was C7orf62, whose function has been unclear. The downregulated gene with the lowest *P* value was* RDH10*, which is essential for synthesis of embryonic retinoic acid and limb, craniofacial, and organ development. The full list of these genes was provided as Supplementary Table 1 in Supplementary Material available online at  http://dx.doi.org/10.1155/2015/164087.

### 3.3. Functional Annotation

**Figure 1:**
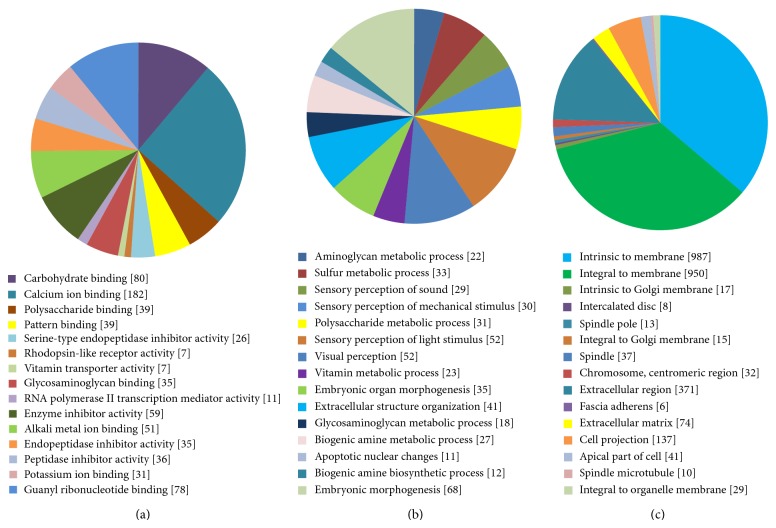
The top 15 enriched GO terms of DEGs. (a) Molecular functions for DEGs (*P* value ≤ 4.61*E* − 03); (b) biological process for DEGs (*P* value ≤ 6.54*E* − 03); (c) cellular component for DEGs (*P* value ≤ 1.95*E* − 03).

To understand the biological roles of the DEGs from pituitary adenomas, we conducted GO categories and KEGG pathway enrichment analysis. GO categories are separated into three groups: biological process, cellular component, and molecular function. We examined GO categories separately. The significantly enriched GO terms for molecular functions were carbohydrate binding (GO: 0030246, *P* = 1.93*E* − 03) and calcium ion binding (GO: 0005509, *P* = 3.10*E* − 03) for molecular functions, while for biological processes they were aminoglycan metabolic process (GO: 0006022, *P* = 9.76*E* − 04) and sulfur metabolic process (GO: 0006790, *P* = 1.07*E* − 03), and for cellular component they were intrinsic to membrane (GO: 0031224, *P* = 2.76*E* − 04) and integral to membrane (GO: 0016021, *P* = 7.43*E* − 04) (Figure 1).

Hypergeometric test with *P* value <0.05 was used as the criteria for pathway detection (Table 3). The most significant pathway in our analysis was neuroactive ligand-receptor interaction (*P* = 3.97*E* − 03). Furthermore, tryptophan metabolism (*P* = 2.42*E* − 02) and cardiac muscle contraction (*P* = 5.38*E* − 02) are also highly enriched.

### 3.4. Protein-Protein Interaction (PPI) Network Construction

**Figure 2:**
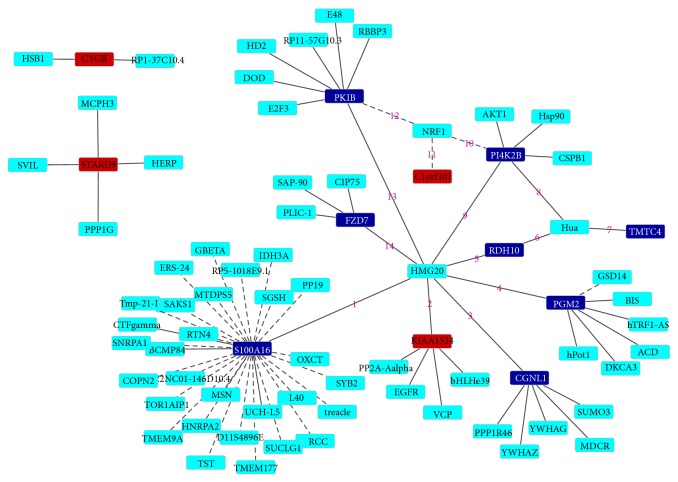
The constructed PPI network for the top 10 up- and downregulated DEGs. Nodes represent proteins; edges represent interactions between two proteins. Red- and blue-color nodes represent products of up- and downregulated DEGs, respectively. Green nodes denote products of genes predicted to interact with the DEGs.

The PPI networks of the top 10 upregulated and downregulated DEGs were established by Cytoscape software including 77 nodes, 76 edges. In the PPI network the nodes with high degree are defined as hub protein, and degrees are defined to measure how many neighbors a node directly connects to. The significant hub proteins in our PPI networks contained S100A16 (Degree = 27), PKIB (Degree = 8), and PGM2 (Degree = 7) (Figure 2).

## 4. Discussion

Pituitary adenomas are monoclonal in origin and most of them are benign, slow-growing neoplasms, and so it is acceptable that many common oncogenes or tumor-suppressor genes which have been confirmed to be involved in human cancers have been indiscoverable in pituitary adenomas. However, advances in molecular biology make it possible to fast-track the search for candidate tumourigenic genes of pituitary adenomas. Some researchers have compared gene expression profiling and identified DEGs between normal human pituitary and pituitary adenomas of different cell origins via microarray technique. Previous microarray studies of human pituitary adenomas have detected many novel candidate genes, including* PTTG* [19],* GADD45* [20],* MEG3a* [21], and* BMP-4* [22].

In this paper, we chose an integrated analysis approach by which 5 microarray datasets were combined to highlight genes that were consistently expressed differentially with statistical significance, performed GO and KEGG pathway enrichment analysis for these genes, and finally constructed PPI networks of the top 10 upregulated and downregulated DEGs. In our integrated analysis, a total of 3994 genes were found to show altered expression in samples of pituitary adenoma compared with NC tissues (2043 upregulated and 1951 downregulated genes). The upregulated gene with the lowest *P* value was* C7orf62*, whose function has been unclear. The downregulated gene with the lowest *P* value was* RDH10*, a member of short chain dehydrogenase-reductase (SDR) family, which is essential for synthesis of embryonic retinoic acid. Additionally* RDH10* was reported to be necessary for limb, craniofacial, and organ development by inducing proliferation arrest, differentiation, and apoptosis [23, 24]. Bankovic et al. found that patients of non-small-cell lung cancer with mutated* RDH10* had shorter survival than those without mutated* RDH10*, confirming its importance in tumor progression. Previous study also identified that* RDH10* played an important role in tumors with lymph node invasion [25]. The role and association with pituitary adenoma have not yet been reported.

In line with previous findings, some genes identified in our integrated analysis have been closely related to the tumorigenesis of pituitary adenomas, such as* GADD45G*,* GADD45B*,* MEG3* [26],* POU1F1* [27],* IGFBP3* [28], and* CCNB1* [29].* GADD45B* and* GADD45G* belong to* GADD45* gene family, and loss of GADD45 expression has been observed in various human cancers [30]. It has been proved that loss of or significantly reduced GADD45G expression is found in most of the pituitary adenomas due to promoter methylation [31]. GADD45B was displayed to be downregulated in pituitary adenomas (-68-fold) through microarray data, subsequently verified by qPCR and immunoblotting, and in vitro experiments identified its role as a tumor suppressor [32]. Cheunsuchon et al. indicated that MEG3 was specifically lost in NFAs, suggesting that its inactivation might be involved in the development of NFAs [33]. The other genes including* POU1F1*,* IGFBP3*, and* CCNB1* were also reported to correlate with pituitary adenomas.

In order to reveal the biological roles of the DEGs from pituitary adenoma, we performed functional annotation for these genes. Neuroactive ligand-receptor interaction, tryptophan metabolism, and cardiac muscle contraction are found to be the top 3 significantly enriched pathways. Many signal transduction pathways are involved in the development of pituitary adenoma including TGF-*β* signal pathway, Wnt signal pathway, and MAPK signal pathway. Interestingly, we noted that the most significant pathway in our analysis was neuroactive ligand-receptor interaction, which is physiologically associated with the neuronal functions. The neuroactive ligand-receptor interaction pathway, which is a collection of neuroactive receptors located on the plasma membranes, is implicated in the stabilization of the neuroendocrine system, indicating that neuroactive ligand-receptor interaction pathway may be involved in the development of pituitary adenoma [34, 35].

Furthermore PPI networks of the top 10 upregulated and downregulated DEGs indicated that the significant hub proteins contained S100A16, PKIB, and PGM2. S100A16, a novel member of S100 protein family which is implicated in cognition in the central nervous system [36], is closely associated with brain pathologies [37]. S100A16 has also been shown to be ubiquitously expressed and upregulated in human tumor [38]. However its function in pituitary adenoma is still unknown for lack of related studies.

The present study has several limitations. First, although global normalization was performed to minimize the heterogeneity of various microarray studies, the heterogeneity cannot be removed completely, which may have distorted the result of analysis. Second, due to limited microarray studies in pituitary adenomas, different subtypes of pituitary adenomas were analyzed, which was not taken into account in our study. Despite these limitations, our findings have important implications for the molecular mechanisms of pituitary adenoma, adding new insights into the future therapy.

In conclusion, by the integrated analysis we have shown the underlying molecular differences between the normal human pituitary and pituitary adenomas of different cell origins and identified DEGs and biological function to contribute to the successful identification of therapeutic targets for pituitary adenoma and the development of effective targeted therapies. Further functional studies may provide additional insights into the role of the differentially regulated genes in the pathophysiology of pituitary adenoma.

## Supplementary Material

The gene symbol, P-value, the average expression value in pituitary adenoma and normal control tissues, and fold change of all DEGs were available in the Supplementary Table 1.

## Figures and Tables

**Table 1:** Characteristics of the individual studies.

GEO ID	Author	Platform	Samples (N : P)	Year
GSE51618	Feng J	GPL6480 Agilent-014850 4x44K G4112F	3 : 7	2013
GSE46311	Lekva T	GPL6244 Affymetrix Human Gene 1.0 ST Array	0 : 16	2013
GSE36314	Oyesiku [10]	GPL8300 Affymetrix Human Genome U95 Version 2 Array	3 : 4	2012
GSE22812	Wierinckx et al. [11]	GPL2895 GE Healthcare/Amersham Biosciences CodeLink Bioarray [11]	0 : 13	2011
GSE4237	Hussaini IM	GPL570 Affymetrix Human Genome U133 Plus 2.0 Array	6 : 4	2006

**Table 2:** The top 10 most significantly up- or down-regulated DEGs.

Gene ID	Gene symbol	Official full name	*P* value	Fold change
Up-regulated genes				
219557	C7orf62	Chromosome 7 open reading frame 62	3.33*E* − 16	2.1819
57519	STARD9	StAR-related lipid transfer (START) domain containing 9	1.28*E* − 14	1.8589
89792	GAL3ST3	Galactose-3-O-sulfotransferase 3	1.29*E* − 13	1.9935
57650	KIAA1524	KIAA1524	1.09*E* − 12	1.351
114757	CYGB	Cytoglobin	4.85*E* − 12	1.8788
157983	C9orf66	Chromosome 9 open reading frame 66	1.73*E* − 11	1.4951
341880	SLC35F4	Solute carrier family 35, member F4	1.91*E* − 11	2.2741
57121	LPAR5	Lysophosphatidic acid receptor 5	3.26*E* − 11	1.3792
257044	C1orf101	Chromosome 1 open reading frame 101	3.63*E* − 11	1.2287
145581	LRFN5	Leucine rich repeat and fibronectin type III domain containing 5	3.80*E* − 11	1.9332

Down-regulated genes				
157506	RDH10	Retinol dehydrogenase 10 (all-trans)	0	−2.3698
8324	FZD7	Frizzled class receptor 7	0	−2.6494
140576	S100A16	S100 calcium binding protein A16	3.00*E* − 15	−1.8248
84952	CGNL1	Cingulin-like 1	2.40*E* − 14	−2.1091
85375	KIAA1661	KIAA1661 protein	4.95*E* − 14	−1.896
55276	PGM2	Phosphoglucomutase 2	7.29*E* − 14	−1.5698
55300	PI4K2B	Phosphatidylinositol 4-kinase type 2 beta	9.64*E* − 14	−1.4987
84899	TMTC4	Transmembrane and tetratricopeptide repeat containing 4	1.20*E* − 13	−1.4509
345557	PLCXD3	Phosphatidylinositol-specific phospholipase C, X domain containing 3	3.01*E* − 13	−3.3246
5570	PKIB	Protein kinase (cAMP-dependent, catalytic) inhibitor beta	1.00*E* − 12	−2.3052

**Table 3:** The enriched KEGG pathway of DEGs.

KEGG pathway	Number of genes	*P* value
Neuroactive ligand-receptor interaction	54	3.97*E* − 03
Tryptophan metabolism	12	2.42*E* − 02
Cardiac muscle contraction	18	5.38*E* − 02
O-Glycan biosynthesis	9	6.01*E* − 02
Taste transduction	13	6.67*E* − 02
TGF-beta signaling pathway	19	7.43*E* − 02
Arrhythmogenic right ventricular cardiomyopathy (ARVC)	17	7.83*E* − 02
Basal cell carcinoma	13	9.46*E* − 02
Colorectal cancer	18	9.56*E* − 02
Amino sugar and nucleotide sugar metabolism	11	9.71*E* − 02
