# Corrigendum to “Identification of Differentially Expressed Genes in Pituitary Adenomas by Integrating Analysis of Microarray Data”

**DOI:** 10.1155/2018/6069189

**Published:** 2018-09-06

**Authors:** Peng Zhao, Wei Hu, Hongyun Wang, Shengyuan Yu, Chuzhong Li, Jiwei Bai, Songbai Gui, Yazhuo Zhang

**Affiliations:** ^1^Department of Neurosurgery, Beijing Tiantan Hospital, Capital Medical University, Beijing, China; ^2^Department of Cardiology, Beijing Chuiyangliu Hospital, Beijing, China; ^3^Beijing Neurosurgical Institute, Center of Brain Tumor, Beijing Institute for Brain Disorders, Capital Medical University, Beijing, China

In the article titled “Identification of Differentially Expressed Genes in Pituitary Adenomas by Integrating Analysis of Microarray Data” [1], there were errors for the control samples in the datasets we selected in the study. These errors were raised by Mateusz Bujko [2].

Dr. Bujko was concerned with the accuracy of the control samples [2] and pointed out the mistake that for the samples in the GSE4237 dataset by Hussaini (2006), the expression profiling data of tumor tissue samples and cell lines (GSM96624, GSM96625, GSM96626, GSM96627, GSM96628, and GSM96629) were mistakenly used as normal controls for the integrative analysis. The aim of this study was to provide clues to the pathogenesis of pituitary adenomas, and we are very willing to try our best to pursue a reliable outcome. Hence, we reviewed the raw experimental records and the bioinformatics analysis workflow to find where the problem might be arising from.

Additionally, we also reanalyzed the data and found that the obtained results were generally identical to those in the published article. The raw datasets, GSE4237, GSE22812, GSE36314, GSE46311, and GSE51618, and the datasets after normalization with Z score are available in the Supplementary Materials. However, we also ran an analysis considering GSM96624, GSM96625, GSM96626, GSM96627, GSM96628, and GSM96629 (from GSE4237) as though they were normal pituitary tissues and found that the obtained genes showing aberrant expression were completely different from those in the published article. Hence, we are certain the inclusion of these samples as normal control samples was just a mistake in writing the article. There was no problem in the data analysis; hence, the results in the published article are reliable. The detail of samples in each dataset were recorded by the author responsible for data analysis (as in Table 1 below), but a lack of communication in preparing the manuscript caused the error that Dr. Bujko pointed out, and the statement that “we obtained a total of 5 expression profiles of pituitary adenoma in GEO database; it contained 44 samples of pituitary adenoma and 12 samples of controls” was inaccurate because only Oyesiku (2012) in GSE36314 and Feng (2013) in GSE51618 directly compared pituitary adenoma and normal tissues. Hence, this integrative analysis contained 50 samples of pituitary adenoma and 6 samples of controls.

Integrative analysis of gene expression profiles in pituitary adenomas will be more valuable compared with small-scale microarray-based expression profiling studies. We hope this integrative analysis will shed light on the pathogenesis of pituitary adenoma. Again, we appreciate Mateusz Bujko's contribution to correcting our mistake in preparing our article.

## Figures and Tables

**Table 1:** Revised characteristics of the individual studies.

GEO ID	Normal : case	Contributor	Year
GSE4237	0 : 10 8 resected human pituitary tumor (1 recurrent + 1 nonrecurrent + 1 invasive + 1 noninvasive + 4 undefined) +2 HP75 tumor cell line	Hussaini IM	2006
GSE22812	0 : 13 Prolactin pituitary tumors (5 noninvasive + 2 invasive + 6 aggressive-invasive)	Wierinckx A	2011
GSE46311	0 : 16 16 somatotroph adenoma (also known as growth hormone-producing somatotroph pituitary adenomas)	Lekva T	2013
GSE36314	3 : 4 4 human prolactinoma 3 human normal pituitary	Oyesiku NM	2012
GSE51618	3 : 7 3 pituitary glands 7 NFPAs (nonfunctioning pituitary adenomas) (4 noninvasive NFPAs + 3 invasive NFPAs)	Feng J	2013

## Supplementary Materials

Supplementary 1 The raw data of the included datasets downloaded from GEO database.

Supplementary 2 The normalized datasets by Z score transformation.
